# Impact of Match Type and Match Halves on Referees’ Physical Performance and Decision-Making Distance in Chinese Football Super League

**DOI:** 10.3389/fpsyg.2022.864957

**Published:** 2022-05-09

**Authors:** Jinying Jiang, Huanmin Ge, Lida Du, Miguel-Angel Gomez, Bingnan Gong, Yixiong Cui

**Affiliations:** ^1^School of Sports Engineering, Beijing Sport University, Beijing, China; ^2^China Football College, Beijing Sport University, Beijing, China; ^3^National Team Administration Department, Chinese Football Association, Beijing, China; ^4^Facultad de Ciencias de la Actividad Física y el Deporte, Universidad Politécnica de Madrid, Madrid, Spain; ^5^Institute of Physical Education and Training, Capital University of Physical Education and Sports, Beijing, China; ^6^AI Sports Engineering Lab, School of Sports Engineering, Beijing Sport University, Beijing, China

**Keywords:** soccer, match context, spatial, fitness, match officials

## Abstract

The purpose of this study was to explore how Chinese Football Super League (CSL) referees’ physical performance and decision-making distance varied according to match type and match halves. Data from 107 matches played by top-4 ranked and bottom-4 ranked teams during 2018–2019 CSL seasons were collected. Level of matches was classified into three groups: (a) upper-ranked (top-4) teams against top-4 teams, (b) top-4 teams against lower-ranked teams (bottom-4), and (c) bottom-4 teams against bottom-4 teams. Two-way ANOVA and Scheirer-Ray-Hare test were used to examine the statistical differences of referees’ physical and spatial related distance variables among different match levels and halves. The Euclidean distance to the ball at the following three variables were statistically different among three match types: clearance (*p* = 0.03, $E_{R}^{2}$ = 0.03), running with the ball (*p* = 0.01, $E_{R}^{2}$ = 0.04), and shot off target (*p* = 0.04, $E_{R}^{2}$ = 0.03). In addition, referees’ distance to the ball at three events were statistically different between both match halves: pass (*p* < 0.001, *r* = 0.69), reception (*p* < 0.001, *r* = 0.76), and running with the ball (*p* < 0.001, *r* = 0.77). The total running distance was statistically different between both match halves (*p* = 0.001, *d* = 0.05). The findings indicated that although CSL referees showed little difference in physical performance when officiating matches of three competitive levels and two halves, distinct rhythms of competitions determined that they needed to adjust running strategies to maintain proper distance to the ball. This study implied that the CSL referees’ match performance was affected by the teams’ style of play and match status.

## Introduction

Football is a team sport mediated by the referees to ensure that players perform under the rules (Hossner et al., 2019; Raab et al., 2020). Helsen and Bultynck (2004) observed that the average number of decision-making for referees was 137 per match in 31 professional male football matches. However, they found that almost 25% of these decisions were incorrect. One of the possible reasons for such phenomenon is that not all the referees executed properly and accurately their jobs within a match (Gulec and Yilmaz, 2016). Consequently, improving the ability to apply the rules during the game is significant for football referees to execute correct decision-making. Another potential reason is related to the referees’ physical preparedness. In order to observe the on-going behaviors of players and execute the rules of a match from a relatively suitable distance and angle, referees must keep appropriate distance to the players and ball and also move quickly and constantly from one area of the football field to another (Ghasemi et al., 2009; Castagna et al., 2013; Gonçalves et al., 2021).

In order to make a more proper decision under the high pressure of a match, referees are required to keep up with the match and to be physically prepared (Slack et al., 2013; Weston, 2015; Joo and Jee, 2019). Although Barbero-Álvarez et al. (2012) found that there was not any difference observed for high-intensity actions between halves for American officials during the 2007 Copa America cup, Castillo et al. (2015) found that the football referees’ physical performance was related to match fatigue and various levels of exercise intensity may cause different degrees of match fatigue to the same person. In particular, lower-ranked teams covered less distance with high-intensity accelerations, while higher-ranked teams covered more distance at high-intensity activities when in possession (Rampinini et al., 2007).

Because of the high level of interactions with players, football officials need outstanding decision-making skills (Schnyder and Hossner, 2016). The best position is one from which the referee can make the correct decision (IFAB, 2020/2021). A longer distance of the referees’ decision-making will increase the risk of missing vital information that should be used as the basis for making a correct decision (Johansen and Erikstad, 2021). Although Johansen and Erikstad (2021) found that referees may take more proper decisions in distances below 10 m than longer distances. A closer decision-making distance may affect the players’ performance and the trajectory of the ball. Hossner et al. (2019) revealed that the referees’ decision-making had been affected by the decision-making position during the FIFA World Cup 2014. Gonçalves et al. (2021) found that the referees maintained a distance of 0–18 m to the ball location in the observed European leagues such as the English Premier League, German Bundesliga, and the Spanish La Liga. Oliveira et al. (2011) found that no significant association was found between the Brazilian referee’s distance from a foul play and the accuracy of the decision-making. However, Mallo et al. (2012) found that the percentage of wrong decision-making of referees would be reduced at a distance of 11–15 m during the 2009 FIFA Confederations Cup.

The previous studies have demonstrated that competition level has an influence on the referees’ physical performance or the performance of the decision-making distance in the top European leagues (Castillo et al., 2018), but little is known about how the Asian referees behave during matches, given that Asian referees are making more international appearance, such as officiating the World Cup and the Asian Cup matches (Tribune, 2018). Furthermore, previous researches have focused on the general decision-making distance of referees instead of different kind of decision-making performance (Hossner et al., 2019). Therefore, it would be essential to understand their physical and different kind of decision-making performance in a football league such as CSL that is less developed and has a slower match rhythm (Zhou et al., 2019), so as to assess their ability and also to determine the milestones for improvement. In light of the abovementioned rationale, this study aimed toward two aspects: (i) to investigate the relationship between the referees’ match performance and the match type; and (ii) to explore the effect of different match halves on the referees’ match performance. The hypothesis was that the CSL referees’ physical performance and decision-making distance would be affected by different match types and match halves. It was expected that the results of the current research would provide useful feedback for refining physical test programs of the CSL referees and help with maintaining proper decision-making distance during on-going behaviors.

## Materials and Methods

### Sample and Data

Data related to the referees’ physical performance and spatial information during 107 CSL matches from 2018 (*n* = 55) and 2019 (*n* = 52) seasons were generated through an optical tracking system by Amisco, whose accuracy, validity, and reliability have been confirmed by previous studies (Randers et al., 2010; Castellano et al., 2014). All 107 matches were completed by the top-4 and bottom-4 teams (teams’ ranking was according to their end-of-season rankings). The number of matches for each team is as follows: top-4 (Shanghai SIPG: 13; Guangzhou Evergrande Taobao: 14; Shandong Luneng Taishan: 7; Beijing Sinobo Guoan: 14; Jiangsu Suning: 7), bottom-4 (Chongqing SWM: 7; Tianjin Teda: 7; Changchun Yatai: 7; Guizhou Hengfeng FC: 7; Tianjin Tianhai: 6; Shenzhen FC: 6; Beijing Renhe: 7; Shanghai Greenland Shenhua: 5).

### Physical and Events-Related Variables

A total of 27 performance indicators (see Table 1) related to the referee’s physical performance and distance to the ball (Castillo et al., 2016; Carvalho et al., 2020; Zhou et al., 2020) within different game events were extracted from the original data. All the decision-making distance-related metrics are awarded in meters. The speed thresholds of physical indicators were defined as: fast speed running (17–21 km/h), high speed running (21–24 km/h), and sprint (24 km/h, +∞), which was provided by Amisco (Randers et al., 2010; Filetti, 2016; Gong et al., 2021). And the sample size related to referees’ decision-making distance are as follows: pass (91,607 observations); goal (358); shot on target (1,089); shot off-target (1,753); reception (64,269); clearance (5,702); direct foul (3,187); out for goal kick (1,765); out for corner (1,122); running with ball (59,714); cross (4,726); foul penalty (37); and offside (449).

**TABLE 1:** Referees match performance indicators and definitions.

Group of indicators	Indicators	Definition
*Physical performance*	Distance (m)	Total distance in the match
	Distance of sprint (m)	Distance covered at sprint speed (24 km/h, +∞)
	Distance of fast speed (m)	Distance covered at fast speed running (17–21 km/h)
	Distance of high speed (m)	Distance covered at high-speed running (21–24 km/h)
	Number of high-speed	Number of high-speed running
	Number of sprinting	Number of sprints
	Average speed (km/h)	Average speed in the match
	Average speed during home possession (km/h)	Average speed when home team has the ball possession
	Average speed during away possession (km/h)	Average speed when away team has the ball possession
	Distance fast running (%)	The proportion of fast running distance in total distance running
	Distance sprint (%)	The proportion of sprinting distance in total distance running
	Distance high speed (%)	The proportion of high-speed running distance in total distance running
	Average length sprint (m)	The average length in a sprint
	Average length high speed (m)	The average length in a high-speed running
*Spatial distance to event*	Goal	Goal achieved
	Pass	An intentional ball played from one player to his teammate
	Clearance	An action (generally a pass) when the player, while having other option, to pass or to hold the ball, is instead clearing it, either with a long pass forward without a precise target or for a throw in/corner kick, playing safe
	Cross	Any pass that delivers the ball into the penalty area by the attacking team, from lateral areas of the attacking third (not played inside of the penalty area)
	Running with ball	Player dribbles and moves together with the ball
	Shot off target	Any clear attempt to score that goes over or wide of the goal without making contact with another player
	Shot on target	An attempt to score a goal which required intervention to stop it going in, or resulted in a goal/shot which would go in without being diverted
	Offside	Being caught in an offside position resulting in a free kick to the opposing team
	Reception	A player goes to catch the ball passed by his teammates
	Out for corner	Ball goes out of play for a corner kick
	Direct foul	A foul that a goal can be scored directly, without the ball being touched by another player
	Foul penalty	A penalty is a foul which occurs within the 18-yard box near a team’s goal, these fouls are only by the defending team against the attacking team within that 18-yard box, if an attacking player is fouled in the box, then that team gets to take a penalty shot on goal
	Out for goal kick	A ball goes over the baseline and is then served by the goalkeeper

### Match Type

Types of matches were classified into the following three groups according to teams’ end-of-season rankings: (i) upper-ranked (top-4) teams facing upper-ranked oppositions; (ii) Top-4 teams facing lower-ranked (bottom-4) teams; and (iii) bottom-4 teams facing lower-ranked teams.

### Statistical Analyses

The assumption of data normality was checked the for all variables using the Kolmogorov–Smirnov test (*p* > 0.05). Subsequently, a two-way ANOVA (Finch, 2005) and the Scheirer-Ray-Hare test (Scheirer et al., 1976; Toothaker and Chang, 1980) were used to examine the statistical differences of the referees’ physical performance among different match types (match contexts) and match halves (*p* < 0.05), with the significance level adjusted following a Scheffe *post-hoc* test and *post-hoc* Mann-Whitney *U* test (Armstrong and Hilton, 2014; MacFarland and Yates, 2016). The effect size estimations for the ANOVA and Scheirer-Ray-Hare tests were computed using partial eta square ($\eta_{p}^{2}$, thresholds of magnitude: small, <0.01; medium, 0.01–0.06; large, 0.06–0.14) and epsilon-squared ($E_{R}^{2}$, thresholds of magnitude: negligible, <0.01; weak, 0.01–0.04; moderate, 0.04–0.16; relatively, 0.16–0.36; strong, 0.36–0.64), respectively (Louis and Parker, 2005; Lakens, 2013; Tomczak and Tomczak, 2014). In addition, the effect size estimations for the Scheffe *post-hoc* tests and *post-hoc* Mann-Whitney *U* tests were computed using Cohen’s *d* (*d*, thresholds of magnitude: trivial, <0.2; small, 0.2–0.6; moderate, 0.6–1.20; large, 1.2–2.0; and very large, >2.0) and the value of the correlation coefficient (*r*, thresholds of magnitude: negligible, <0.1; weak, 0.1–0.39; moderate, 0.39–0.69; strong, 0.69–0.89; and very strong, >1.00) (Hopkins et al., 2009; Schober et al., 2018). The *r* is calculated using the following equation (Tomczak and Tomczak, 2014):

$$r=\frac{\text{Z}}{\sqrt{n}}$$

where *Z* standardized value for the *U-*value, *n* is the total number of observations.

After the statistical analysis, to verify whether the referees’ match performance was related to the goals scored in both halves, a density distribution of match goal time for each half was presented and a Chi-square test was used for the number of goals during the first half and the second half of the CSL to help further clarify the difference in match goal timeline for the CSL teams between the first half and the second half. Effect sizes of the tests were calculated using the Cramer’s V and their interpretation was based on the following criteria: <0.10, small; 0.10–0.30, medium; and 0.30–0.50, and large (Volker, 2006). All the analyses were done using Python 3.7.

## Results

The descriptive statistics of all performance indicators and the results of the interaction between match type and match halves were shown in Tables 2, 3. The Supplementary Tables 1–4 shows the specific results of two main effects. No statistical difference was identified in the interaction between match type and match halves. The two-way ANOVA showed that only total running distance was statistically different between different match halves (*p* < 0.01, *d* = 0.05). There are three indicators (clearance: *p* = 0.03, $E_{R}^{2}$ = 0.03; running with ball: *p* = 0.01, $E_{R}^{2}$ = 0.04; and shot off target: *p* = 0.04, $E_{R}^{2}$ = 0.03) statistically different among different match contexts, as shown by the Scheirer-Ray-Hare test. While pass (*p* < 0.01, *r* = 0.69), reception (*p* < 0.01, *r* = 0.76), and running with ball (*p* < 0.01, *r* = 0.77) were statistically different between the first and the second half.

**TABLE 2:** Results of two-way ANOVA between match type and a half period.

Variable	M (SD)	F	*p*	$\eta_{p}^{2}$ (ESI)
Distance (m)	3,229.04 (248.05)	1.61	0.20	0.015 (L)
Average speed during home possession (km/h)	5.27 (0.50)	1.71	0.18	0.016 (L)
Distance high speed (m)	49.3 (22.33)	0.01	0.99	0.000 (S)
Distance fast running (m)	163.65 (50.57)	0.16	0.85	0.002 (S)
Distance fast running (%)	5.02 (1.37)	0.03	0.97	0.000 (S)
Foul penalty	15.38 (5.21)	0.28	0.76	0.017 (L)

*ESI, effect size interpretation; S, small; M, medium; L, large.*

**TABLE 3:** Results of Scheirer-Ray-Hare test between match type and a half period.

Variable	M (SD)	H	*p*	${\text{E}}_{R}^{2}$ (ESI)
Average speed (km/h)	3.98 (0.31)	0.98	0.61	0.009 (N)
Average speed during away possession (km/h)	5.28 (0.52)	0.52	0.77	0.005 (N)
Number sprint	0.96 (0.76)	0.05	0.97	0.0005 (N)
Distance sprint (m)	20.88 (18.20)	0.12	0.94	0.001 (N)
Distance sprint (%)	0.64 (0.56)	0.11	0.95	0.001 (N)
Average length sprint (m)	21.13 (9.44)	1.06	0.59	0.001 (N)
Number high speed	3.19 (1.38)	0.08	0.96	<0.000 (N)
Distance high speed (%)	1.51 (0.65)	0.07	0.97	0.001 (N)
Average length high speed (m)	15.55 (2.92)	0.02	0.99	<0.000 (N)
Clearance	16.13 (6.14)	0.84	0.43	<0.000 (N)
Cross	23.20 (9.64)	0.29	0.75	<0.000 (N)
Pass	18.96 (9.08)	0.97	0.38	<0.000 (N)
Reception	19.57 (8.58)	2.03	0.13	<0.000 (N)
Running with ball	17.84 (7.77)	1.14	0.32	<0.000 (N)
Shot off target	12.24 (5.35)	0.34	0.71	<0.000 (N)
Shot on target	13.87 (5.53)	1.35	0.26	0.001 (N)
Foul direct	14.58 (6.20)	1.69	0.18	0.001 (N)
Out for corner	22.35 (5.86)	0.55	0.58	0.001 (N)
Out for goal kick	24.65 (6.63)	1.23	0.29	0.001 (N)
Goal	21.46 (4.48)	0.66	0.52	0.002 (N)
Offside	17.61 (6.33)	0.65	0.52	0.001 (N)

*ESI, effect size interpretation; N, negligible; W, weak; M, moderate; R, relatively; S, strong.*

**FIGURE 1:**
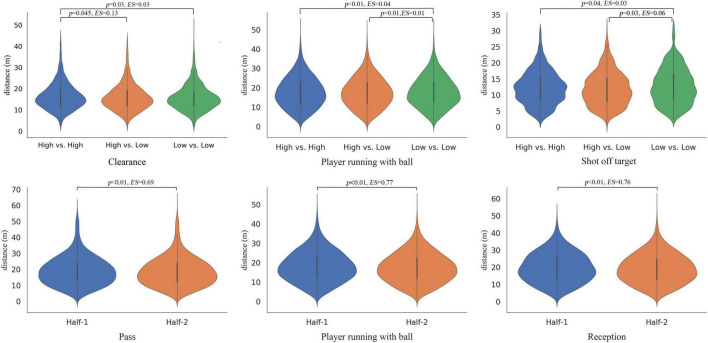
Referees’ decision-making distance (m) within different game events. Violin plots showing the distribution of the computed variables based on match levels and match periods. These violin plots combine box plots indicating first and third quartiles and vertical lines 10th and 90th percentiles. Referees’ decision-making distance for match type (top row) and match period (bottom row).

Figure 1 shows the distribution of the indicators with statistical difference on different main effect. The CSL referees kept a closer distance during the low vs. high matches when events were players running with the ball (17.76 ± 7.77 m) and shot off-target (12.00 ± 5.35 m). While officiating the low vs. low teams matches, they maintained a closer distance when the event was clearance among three-match levels (15.91 ± 5.35 m). They also kept a closer distance to the ball in the second half when the event was a pass (18.71 ± 9.06 m), the player running with the ball (17.48 ± 7.73 m), and reception (19.25 ± 8.49 m). After the *post-hoc* test for different match levels, the results showed that referees’ distance to clearance (*p* = 0.045, *r* = 0.13) was statistically higher while referring top-4 vs. top-4 teams matches (16.55 ± 6.14 m) than top-4 vs. bottom-4 teams matches (16.07 ± 6.13 m). The distance to a player running with the ball (*p* = 0.01, *r* = 0.01) was statistically lower when officiating top-4 vs. bottom-4 teams matches (17.76 ± 7.77 m) than bottom-4 vs. bottom-4 teams matches (18.03 ± 7.77 m), and also shot off-target (*p* = 0.03, *r* = 0.06, top-4 vs. bottom-4 teams matches (12.00 ± 5.35 m), and bottom-4 vs. bottom-4 teams matches (12.87 ± 5.35 m).

**FIGURE 2:**
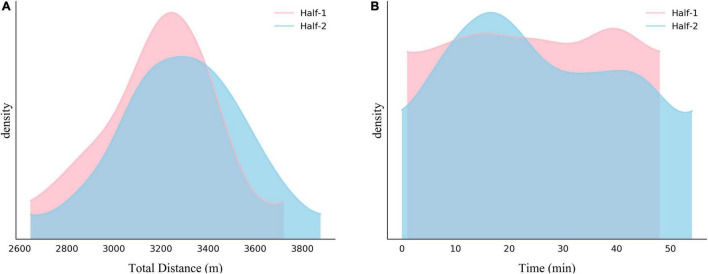
Density distribution of referees’ running distance **(A)** and goal time **(B)** during each half of the match.

Figure 2A shows the density distribution of referees running distance for each half. More than 80% of CSL referees’ running distance in one half is between 2,800 and 3,700 m. The values of total running distance sightly increased from the first half to the second half (*p* = 0.001, *d* = 0.048). The density distribution of match goal time for each half can be seen in Figure 2B. Compared to the goal time line in the first half, more goals happened during the 10–30 min of the second half (χ^2^ = 0.11, *p* = 0.74, and Cramer’s *V* = 0.02). Overall, more goals were scored in the second half (*n* = 197) than during the first half (*n* = 161).

## Discussion

This is the first study exploring referees’ physical performance and decision-making distance considering the match type and match halves during the CSL. The main findings showed that both factors did not interact with the effects of referees’ match performance. However, the CSL referees ran more and kept a closer decision-making distance during the second half. In addition, they kept a closer decision-making distance during events of shot off target and player running with the ball when refereeing high-ranked teams against low-ranked teams, and during events of clearance when refereeing low-ranked teams vs. high-ranked teams. No interaction was found in the physical indicators and decision-making distance among different match types and halves, which implied that the CSL referees kept the same decision-making level no matter in different match contexts or in different match halves. However, the results of the main effect for match type and match halves confirmed the hypothesis that the CSL referees’ physical performance and decision-making distance would be affected by different match types and match halves.

Referees kept a closer distance while refereeing the low-ranked teams against the high-ranked teams when events were players running with the ball and shot off target. Such findings may reveal that the referees’ performance was affected by the teams’ style of play, as well as their competitive levels, which is in accordance with the findings of Stolen et al. (2005). On the one hand, referee bias in favor of successful teams (Erikstad and Johansen, 2020) (which is likely to be the higher-ranked teams) would cause the different decision-making distance when referees meditated the high-ranked teams vs. low-ranked teams. On the other hand, a recent study pointed out that weak teams play more quick counterattacks and top teams are adopted a possession style of play (Gómez et al., 2018). Then, to anticipate the play and achieve the best position to keep up with the match pace (Raab et al., 2020), referees logically adopt a different decision-making distance when events were players running with the ball and shot off target. Compared with the stronger opponents, low-ranked teams were shown to underperform during established defense (Gollan et al., 2018) and maintain the lower percentage of ball possession, which in turn provides their opponents a better chance to attack the goal. Therefore, to create a better angle to move at the same pace as match-play, referees maintained a closer distance to the low-ranked teams against high-ranked teams when the event was a clearance.

A closer synchronized distance and more running distance in the second half were performed by the CSL referees, which is not in line with Mallo et al. (2009) and Weston et al. (2011a) in their analysis of English Football Association Premier League referees. The result possibly implies that the referees’ match performance was influenced by different half periods and the familiarity with the confronting teams. A possible explanation could be the influence of the players’ performance on that of referees (Weston et al., 2008, 2011b). Previous studies found that winning status induced a greater total distance in elite football players (Moalla et al., 2017), and such phenomenon will further differentiate the referees’ physical performance between match periods (Weston et al., 2008, 2011b). The current research found that more goals were scored in the second half of the CSL. In order to recognize ongoing competitive behaviors, referees meditated a closer synchronized distance to the ball, which might increase their running distance in the second half. On the other hand, their performance was also influenced by tactical-technical behaviors (USSF, 2018), as the current findings clearly pointed out that the CSL referees’ running distance would be susceptible to critical events such as goals and reception. Hence, referees need to be adapted to changes in the players and team tactics.

In a word, the match type and match period had affected the CSL referees’ match performance, which was evidenced by the referees’ increased running distance in the second half and different decision-making distance under three match types. The match period was an important factor that affected the referees’ match performance, and a closer distance to the ball within various match events and more total running distance in the second half would make referee become more synchronized with a match. Therefore, in order to meditate behaviors of players from a better angle and stay in an area where they are required to stay highly attentive (IFAB, 2020/2021), referees should choose a different running strategy to drop in the right place at the right time. Meanwhile, fitness is a key factor for referees being able to get the right place, and the positioning is the second deciding whether they reach the right place (USSF, 2018). They jointly decide on a good decision-making distance and angle. Therefore, in order to move to the next phase of play and create the best angle of vision, the referees should learn to anticipate the play and integrate the cognitive practice into their fitness training (Gulec and Yilmaz, 2016).

Although the study has provided empirical evidence related to the referees’ physical and decision-making performance under different match types and halves, there are several limitations needed to be acknowledged. First of all, a subjective classification of match type had been adopted in this study. However, a team’s end-of-season ranking only partially represents its real strength (Li et al., 2020), so that such classification omit those middle-ranked teams that were either as competent as high-ranked teams, or as underperformed as low-ranked teams. Second, as football match performance is usually influenced by other contextual factors such as match location and match score-line (Black et al., 2019), the failure to consider them would limit the interpretation of the current findings. Furthermore, the spatial–temporal relationships between referees and players, and their relative angle with match events are important aspects the study was unable to include, so that future research is suggested to extend the current study design, integrating more performance constraints.

## Conclusion

Although CSL referees showed little difference in physical performance when officiating three types of matches as well as two match halves, distinct ranks of teams determined that they need to adjust running strategies so as to maintain proper distance to the ball. In order to adjust better decision-making distance in matches, they also need to change their running strategies in different half periods. As for the practical applications, while preparing for the upcoming match, referees should take the initiative to understand the playing styles of the involved teams so as to know what kind of match is expected to happen and also the corresponding actions they particularly need to take; meanwhile, the decision-making distance in this study could be used as an important reference for novice and sub-elite referees when training and officiating.

## Data Availability Statement

The datasets generated for this study are available on request to the corresponding author.

## Author Contributions

All co-authors equally contributed to the manuscript. JJ designed the experiments and wrote the draft of the manuscript. LD and BG collected and provided the raw data. HG and M-AG provided part of the manuscript revision and contributed to the final draft. YC contributed to the introduction, rationale, and discussion of the main findings, supervised the final draft of the document and revision process, and provided the funding for the study. All authors approved the submitted version.

## Conflict of Interest

The authors declare that the research was conducted in the absence of any commercial or financial relationships that could be construed as a potential conflict of interest.

## Publisher’s Note

All claims expressed in this article are solely those of the authors and do not necessarily represent those of their affiliated organizations, or those of the publisher, the editors and the reviewers. Any product that may be evaluated in this article, or claim that may be made by its manufacturer, is not guaranteed or endorsed by the publisher.
