# Supplementary material for: Impact of Match Type and Match Halves on Referees’ Physical Performance and Decision-Making Distance in Chinese Football Super League
Source: Front Psychol. 2022 May 9;13:864957. doi: 10.3389/fpsyg.2022.864957 (PMC9125187; doi:10.3389/fpsyg.2022.864957)
Supplement: Supplementary file 1 [file Table_1.docx]

| Supplementary Table 1. Main effect of Two-way ANOVA for the *match type* | | | |
| --- | --- | --- | --- |
| Indicator | F | *p* | $\eta_{p}^{2}$ (ESI) |
| Distance (m) | .05 | .95 | <.000 (S) |
| Av speed during home possession (km/h) | 0.99 | .37 | .009(S) |
| Distance high speed (m) | .62 | .54 | .006 (S) |
| Distance fast running (m) | .23 | .79 | .002 (S) |
| Distance fast running (%) | .24 | .79 | .002 (S) |
| Foul penalty | 1.01 | .38 | .061 (M) |
| Note: ESI= effect size interpretation; S= small; M= medium; L= large. | | | |

| Supplementary Table 2. *Main effect of* Scheirer-Ray-Hare test *for the match type* | | | |
| --- | --- | --- | --- |
| Indicator | H | *p* | $E_{R}^{2}$ (ESI) |
| Av speed (km/h) | .13 | .94 | .001 (N) |
| Av Speed during away possession (km/h) | .28 | .87 | .003 (N) |
| Number sprint | 1.48 | .48 | .014 (N) |
| Distance sprint (m) | 1.79 | .41 | .017 (N) |
| Distance sprint (%) | 1.67 | .43 | .016 (N) |
| Average length sprint (m) | .93 | .63 | .009 (N) |
| Number high speed | 1.54 | .46 | .015 (N) |
| Distance high speed (%) | .48 | .79 | .005 (N) |
| Average length high speed (m) | 1.97 | .37 | .019 (N) |
| Clearance | 3.45 | .03 | .030 (N) |
| Cross | 2.44 | .09 | .020 (N) |
| Pass | .49 | .61 | .005 (N) |
| Reception | .97 | .38 | .009 (N) |
| Running with ball | 4.53 | .01 | .040 (W) |
| Shot off target | 3.16 | .04 | .030 (N) |
| Shot on target | 1.51 | .22 | .010 (N) |
| Foul direct | .03 | .97 | .000 (N) |
| Out for corner | 2.55 | .08 | .020 (N) |
| Out for goal kick | 1.38 | .25 | .010 (N) |
| Goal | 1.66 | .19 | .020 (N) |
| Offside | .24 | .79 | .008 (N) |
| Note: ESI= effect size interpretation; N= negligible; W= weak; M= moderate; R= relatively; S= strong. | | | |

| Supplementary Table 3. *Main effect of* Two-way ANOVA *for the match period* | | | |
| --- | --- | --- | --- |
| Indicator | F | *p* | $d$ (ESI) |
| Distance (m) | 10.53 | .001 | .048 (S) |
| Av speed during home possession (km/h) | .004 | .95 | .000 (T) |
| Distance high speed (m) | .54 | .47 | .003 (T) |
| Distance fast running (m) | 3.8 | .05 | .018 (T) |
| Distance fast running (%) | 1.73 | .19 | .008 (T) |
| Foul penalty | 3.05 | .09 | .090 (M) |
| Note: ESI= effect size interpretation; S= small; T= trivial; M= medium; L= large. | | | |

| Supplementary Table 4. *Main effect of*Scheirer-Ray-Hare test *for the match period* | | | |
| --- | --- | --- | --- |
| Indicator | H | *p* | r (ESI) |
| Av speed (km/h) | 1.64 | .20 | .016 (N) |
| Av speed in away possession (km/h) | .08 | .77 | .001 (N) |
| Number sprint | .67 | .41 | .006 (N) |
| Distance sprint (m) | .24 | .62 | .002 (N) |
| Distance sprint (%) | .46 | .50 | .004 (N) |
| Average length sprint (m) | .01 | .93 | <.000 (N) |
| Number high speed | .46 | .50 | .004 (N) |
| Distance high speed (%) | .06 | .81 | .001 (N) |
| Average length high speed (m) | .15 | .7 | .001 (N) |
| Clearance | .30 | .59 | .003 (N) |
| Cross | 3.35 | .07 | .030 (N) |
| Pass | 73.95 | <.000 | .690 (M) |
| Reception | 81.76 | <.000 | .760 (M) |
| Running with ball | 81.82 | <.000 | .770 (M) |
| Shot off target | .05 | .83 | .000 (N) |
| Shot on target | .10 | .76 | .001 (N) |
| Foul direct | .05 | .83 | .000 (N) |
| Out for corner | .37 | .54 | .003 (N) |
| Out for goal kick | 1.53 | .22 | .010(N) |
| Goal | .12 | .74 | .001 (N) |
| Offside | .15 | .70 | .001 (N) |
| Note: ESI= effect size interpretation; N= negligible; W= weak; M= moderate; S= strong; V= very strong. | | | |
